# Evaluation of the Efficacy of Dexamethasone-Eluting Electrode Array on the Post-Implant Cochlear Fibrotic Reaction by Three-Dimensional Immunofluorescence Analysis in Mongolian Gerbil Cochlea

**DOI:** 10.3390/jcm10153315

**Published:** 2021-07-28

**Authors:** Philippine Toulemonde, Michaël Risoud, Pierre Emmanuel Lemesre, Cyril Beck, Jean Wattelet, Meryem Tardivel, Juergen Siepmann, Christophe Vincent

**Affiliations:** 1 Department of Otology and Neurotology, CHU Lille, University of Lille 2 Henri Warembourg, F-59000 Lille, France; michael.risoud@chru-lille.fr (M.R.); pierreemmanuel.lemesre@chru-lille.fr (P.E.L.); cyril.beck90@gmail.com (C.B.); wattelet59@hotmail.fr (J.W.); juergen.siepmann@univ-lille.fr (J.S.); christophe.vincent@chru-lille.fr (C.V.); 2 INSERM U1008—Controlled Drug Delivery Systems and Biomaterials, F-59000 Lille, France; 3 BioImaging Center Lille-Nord de France (BICeL), University of Lille 2 Henri Warembourg, F-59000 Lille, France; Meryem.tardivel@univ-lille.fr

**Keywords:** auditory implant, dexamethasone, hearing loss, rehabilitation, clearing, imaging, light-sheet microscopy

## Abstract

Cochlear implant is the method of choice for the rehabilitation of severe to profound sensorineural hearing loss. The study of the tissue response to cochlear implantation and the prevention of post-cochlear-implant damages are areas of interest in hearing protection research. The objective was to assess the efficacy of dexamethasone-eluting electrode array on endo canal fibrosis formation by three-dimensional immunofluorescence analysis in implanted Mongolian gerbil cochlea. Two trials were conducted after surgery using Mongolian gerbil implanted with dexamethasone-eluting or non-eluting intracochlear electrode arrays. The animals were then euthanised 10 weeks after implantation. The cochleae were prepared (electrode array in place) according to a 29-day protocol with immunofluorescent labelling and tissue clearing. The acquisition was carried out using light-sheet microscopy. Imaris software was then used for three-dimensional analysis of the cochleae and quantification of the fibrotic volume. The analysis of 12 cochleae showed a significantly different mean volume of fibrosis (2.16 × 10^8^ μm^3^ ± 0.15 in the dexamethasone eluting group versus 3.17 × 10^8^ μm^3^ ± 0.54 in the non-eluting group) (*p* = 0.004). The cochlear implant used as a corticosteroid delivery system appears to be an encouraging device for the protection of the inner ear against fibrosis induced by implantation. Three-dimensional analysis of the cochlea by light-sheet microscopy was suitable for studying post-implantation tissue damage.

## 1. Introduction

Sensorineural hearing loss is characterised by the loss of hair cells and by degeneration of the spiral ganglion neurons [1,2]. Cochlear implantation is used as the surgical solution for the rehabilitation of severe to profound sensorineural hearing loss [3,4]. Despite its high success rate, cochlear implantation is responsible for cellular destruction, and inflammatory response with the creation of fibrosis and endo-cochlear neo-osteogenesis [5,6,7,8,9,10,11,12]. The extent of this fibrosis correlates with residual hearing loss where it exists [10,13]. It has also been shown that the increase of implant impedances is directly related to the extent of the fibrotic reaction around the electrode array [14]. This necessitates increasing the intensity of the electrical stimulus. Electrical stimulation may have detrimental effects on the auditory hair cells and spiral ganglion neurons [15,16]. In addition, in patients with residual hearing, it is important to preserve it to allow rehabilitation by combined electrical and acoustic stimulation cochlear implant devices [17,18].

Several measures to reduce surgical trauma are used in current clinical practice with improved surgical techniques, such as electrode arrays that have smaller diameters and greater flexibility and the administration of systemic or trans-tympanic corticosteroid therapy [17,19,20,21,22,23,24,25,26]. Indeed, corticosteroids are mainly studied because of their cell-protective properties against local or sound trauma [27,28,29]. However, the cochlea is protected from the rest of the body by the labyrinthine barrier, which limits the delivery of drugs to the inner ear and exposes the body to adverse effects [30,31]. Thus, for several years, numerous devices have been developed for the local delivery of drugs to the inner ear [32,33,34,35]. Drug delivery via the electrode array using the implant itself as a carrier has been the most attractive option at present to reduce the adverse effects associated with implant trauma [14,36].

The study of post-implantation cochlear damage (cell survival and fibrosis formation) is made difficult by the otic capsule surrounding the entire cochlea and its helical structure. Confocal laser scanning microscopy provides a three-dimensional (3D) representation of a sample by constructing a stack of 2D serial slices, referring to optical sections in confocal planes. Its initial use has already allowed good resolution of the organ of Corti in 3D and thus cell counting [37,38,39]. However, dissection of this structure destroys the surrounding structures, and the use of confocal microscopy is limited to small volume samples [37,40]. Furthermore, the analysis of the scala tympani and therefore of the endo canal fibrosis is not allowed. Indeed, this study requires serial cochlear sections and 3D reconstruction which are time consuming and responsible for a loss of membrane information and architectural deformations of the organ of Corti [41,42,43,44,45]. Most importantly, serial sections of embedded implanted cochlea require the electrode array to be removed, which disrupts the tissue response. Light-sheet microscopy is a technique of choice for studying biological structures in three dimensions. It allows three-dimensional analysis of whole cochlear samples without dissection trauma and leaving the cochlear implant in situ during acquisition in order to quantify the volume of fibrosis around the implant [46,47]. Although the resolution is not as good as with confocal microscopy, cellular evaluation is also possible with immunostaining of hair cells, neurons, and fibrosis [46,47]. These imaging investigations (confocal microscopy and light sheet) require transparent samples. With the development of these imaging techniques, numerous clearing protocols have been developed in recent years and adapted to cochlear clarification [46,47,48].

The objective of this study was to assess the efficacy of the dexamethasone-eluting electrode array on endo canal fibrosis formation by three-dimensional immunofluorescence analysis of the implanted Mongolian gerbil cochlea.

## 2. Materials and Methods

The study was approved by the regional committee for experimental animal care and use (CEEA Nord-Pas de Calais n°75, Lille, France) listed under protocol 2017071021362273. Our work was conducted in agreement with the standard guidelines of the French Ministry of Agriculture, according to the EU Directive 2010/63/EU for the protection of animals used for scientific purposes. The Mongolian gerbils were implanted in a round window on the right side with an electrode array of two electrodes. The left cochleae were left as controls, not implanted. Two trials were conducted after surgery using Mongolian gerbil implanted with dexamethasone-eluting (10%) or non-eluting intracochlear electrode arrays. A CT scan (MicroTEP-CT Inveon; Siemens, Munich, Germany) was performed at 5 and 10 weeks after implantation to ensure stability and correct positioning of the electrode array. The animals were then euthanised at 10 weeks after implantation and both cochleae (right implanted and left control) were collected.

### 2.1. Electrode Array

For this study, we used prototype implants supplied by Oticon Medical (Vallauris, France) which consisted of 3 parts. An electrode array whose silicone was loaded with dexamethasone or not. It included 2 stimulus-sensing electrodes with a surface area of 0.12 mm^2^ (0.4 mm length × 0.3 mm diameter). Each electrode consisted of a wire made of 90% platinum and 10% iridium. This was the part inserted in a round window at a depth of approximately 3 mm. There was also the ground electrode which was placed subcutaneously, at the level of the contralateral auditory bulla. Additionally, the connector, which was accessible on the animal’s vertex after surgery, allowed the connection of the implant’s electrodes to the various stimulus detection and electrophysiological measurement platforms.

### 2.2. Cochlear Implant Surgery

The procedure was performed under general anaesthesia using a MINITAG veterinary gas anaesthesia station (Tem Sega, Pessac, France). A mixture of air (2 L/min) and isoflurane (5%) was used for induction, and maintenance was achieved with 0.8 L/min of air and 1.5–2% isoflurane (Aerrane; Baxter, Deerfiel, IL, USA). An injection of Buprenorphine (Bupaq 0.3 mg/mL; Virbac, Carros, Carros, France) at a dosage of 0.1 mg/kg was performed 45 min before the incision. Lidocaine (Xylocaine spray 5%; Astrazeneca, London, UK) was used at the incision site to optimise local pain control. All procedures were performed by the same operator after a period of training of a few months. A small right retroauricular incision was made and the muscles covering the bulla were retracted. The anterior portion (anterior to the upper pillar) of the bulla was opened with micro forceps to expose the round window which was then clearly visible. The left auditory bulla was approached in the same way with an anterior and posterior opening of the bulla to expose the lateral semicircular canal which was cut to create a labyrinthine fistula for easier electrophysiological measurements. The connector was then placed on the animal’s vertex. The skin of the vertex was incised vertically down to the galea which was removed with hydrogen peroxide. Two drill holes were made with a 1.18 mm diameter cutter on either side of the sagittal suture. Self-tapping screws were inserted into the drill bits to attach the glue and surgical cement. The connector was then fixed with glue (super bond, SunMedical, Le Havre, France, Réf: 12–900) and surgical cement (Unifast Trad, Réf: 339114, 339292, Phymep, Paris, France), which was applied to the surface of the connector adherent to the vertex. Tunnelling was performed under the muscle, from the vertex incision to the left auditory bulla, to place the ground electrode, and the procedure was repeated on the right side. The right retro auricular approach was then repeated. A notch in the bony rim of the round window was made, paying attention to avoid the stapedial artery located at the upper part of the round window. The window was then opened and the electrode array was carefully inserted. An inert, immediately curing silicone (Kwick sil, world precision instrument, Sarasota, FL, USA) was instilled into the auditory bulla to allow the cochlear implant to be maintained. The incisions were then sutured in a skin plane.

### 2.3. Tissue Preparation

For this procedure, 10 weeks after implantation, the right auditory bulla was again approached under general anaesthesia to cut the electrode array at the entrance to the round window to avoid the risk of explanting the animal, and then the gerbils were euthanised by cervical dislocation. This procedure was performed under gas anaesthesia (gas mixture of air (2 L/min) and isoflurane (5%). The right and left cochlea were then collected. Perfusion of 4%paraformaldéhyde solution was performed through the round and oval windows and the cochleae were placed in 4% paraformaldéhyde solution overnight. The cochleae were then rinsed 3 times for 10 min, with phosphate-buffered saline (PBS 1X) at pH 7.4 with agitating before being decalcified. Decalcification was carried out with 10% EDTA (ethylene diamine tetra-acetic acid) pH 7.26 for 20 days with a change of solution every 48 h. All these steps were carried out at room temperature. Excess bone from the decalcified cochlea was then removed and a small puncture was made at the apex with a 26 Gauge needle to allow diffusion of antibodies.

The immunolabelling protocol required permeabilisation with dimethylsulphoxid (DMSO) twice for 10 min followed by washing 3 times for 20 min in 1X PBS. These steps were carried out at room temperature with agitation. The samples were then placed in a blocking solution (PBS-GT: 1% triton X-100, and 0.2% gelatine from cold-water fish skin in 1X PBS) overnight and then incubated in the primary antibody solution (in 1 mL of PBS-GT) for 3 days. Alpha SMA (Monoclonal mouse IgG2a antismooth muscle actin; Sigma; Saint Quentin Fallavier, France, ref: A2547, concentration 1:400) was used for labelling fibroblasts. NakATPase alpha 3 (Monoclonal mouse IgG1 anti-NaKATPase alpha 3; Invitrogen, Fischer Scientific, Illkirch, France, ref: MA3-915; concentration 1:400) was used for labelling spiral ganglion type 1 afferent neurons. F4/80 (Monoclonal rat IgG2a anti F4/80; Invitrogen; ref: MF48000; concentration 1:200) was used for macrophage labelling. After incubation of the primary antibodies, the samples were rinsed in PBS-T 3 times for 10 min and then incubated in the secondary antibody solution (in 1 mL of PBS-GT) overnight (Goat anti-rat IgG Alexa fluor 488, ref: A-11006, Invitrogen; Goat anti-mouse IgG2a Alexa fluor 647, ref: A-21241, Invitrogen; Goat anti-mouse IgG1 Alexa fluor 594, ref: A-21125, Invitrogen). The concentration used for secondary antibodies was 1:500. The cochleae were again washed 3 times for 10 min in PBS-T. All of these steps were performed at 37 °C, with agitation. The tissue was rinsed twice for 10 min in 1X PBS with agitation at 4 °C. The volumes of the washing solution were 20 mL. The cochleae were then placed in different dilutions of ethanol solutions with a pH > 9.0 (dilution range: 50%, 70%, 100%, 100%) for 12 h each, with agitation at 4 °C. It is important to note that injectable water was used for these dilutions in order to keep the pH stable. At the end of the process, the cochleae were transferred to ethyl cinnamate overnight at room temperature. From the incubation of the secondary antibodies, the steps were carried out in the dark.

### 2.4. Light-Sheet Microscopy

We used an ultramicroscope (La Vision Biotec GmbH, Bielefeld, Germany) for the acquisition of light-sheet microscopy. The objectives used for the acquisitions were a 4×/0.3 and a 20× (La Vision Biotec). Laser diodes (wavelengths 488, 561, and 650) were used to generate the light sheet. The emitted signal was then detected by a Neo sCMOS camera (Andor, Oxford Instruments, Belfast, UK). We set the laser exposure time to 200 ms. The step size was set to 3 µm. The acquisition time was approximately 10 min per laser wavelength. The acquisition software was Inspector Pro 285 (La Vision Biotec). The cochlea was immersed in our clearing medium (ethyl cinnamate) and was held immobile by a perpendicular screw, apex facing the light source.

### 2.5. Three-Dimensional (3D) Analysis

The acquired images (.tiff format) were then converted to an Imaris file (.ims format) using the Imaris file converter (v9.2.1, Oxford Instruments, Abingdon, UK) to allow viewing and processing of the images in 3D with Imaris software (v9.2.1, Oxford Instruments, Abingdon, UK). To quantify the fibrotic volume (in µm^3^) around the implant, we used a creation wizard available with the Imaris software. To quantify the fibrotic volume around the electrode array, we used an automatic creation surface creation wizard available in the Imaris software. This wizard was based on the color and intensity of the source channel. The first step was to define the region of interest of the image and to select the source channel of the surface to create. The absolute intensity threshold value was selected. The smooth option was selected to reduce the noise. Once the object was created, all its statistical values including the volume were found in the statistics tab available on the Imarid software. The creation parameters were saved and reused for all the acquisitions to allow an identical analysis.

### 2.6. Statistical Analysis

Statistical analysis was performed using Excel and SPSS version 19 (IBM, Armonk, NY, USA) software. The Shapiro–Wilk test was used to test the normality of the variables. The comparison of means between independent groups was performed with the Wilcoxon–Mann–Whitney test.

## 3. Results

**Figure 1:**
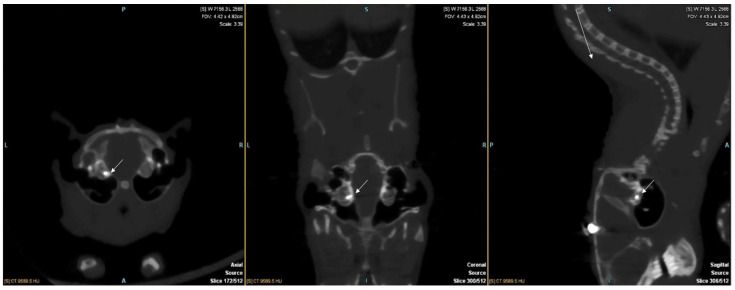
Axial, coronal, and sagittal slice reconstruction of a gerbil’s CT scans at 10 weeks after implantation. The 2 electrodes are visualised in the right cochlea (arrows).

In total, 12 gerbils were implanted. CT scans at 5 weeks and 10 weeks (before euthanasia) confirmed good implantation of all the animals (Figure 1).

**Figure 2:**
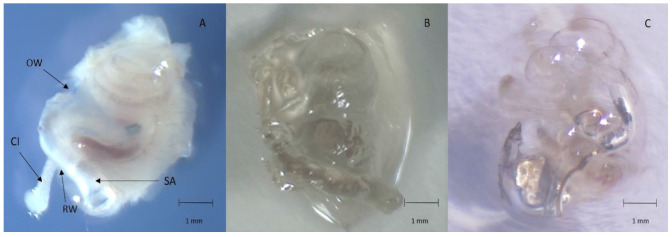
(**A**): fixed and decalcified right cochlea (OW: oval window; RW: round window; SA: stapedial artery; CI: cochlear implant); (**B**): transparent right cochlea outside of the clearing medium; (**C**): transparent right cochlea immerged in the clearing medium (the electrode array in the basal turn of the cochlea is clearly visible). Scale: 1 mm.

The 12 implanted cochleae were prepared following the protocol described above. Decalcified and then cleared gerbil cochleae were completely transparent with an intracochlear electrode array in place (Figure 2). The architecture and size of the cochleae were respected (Figure 2). The orientation of the cochlea allowed good visualisation of the whole cochlea. The laser power was kept low (20%) with an exposure time of 200 ms, to avoid bleaching of the samples. No opacification of the samples was observed during acquisition.

**Figure 3:**
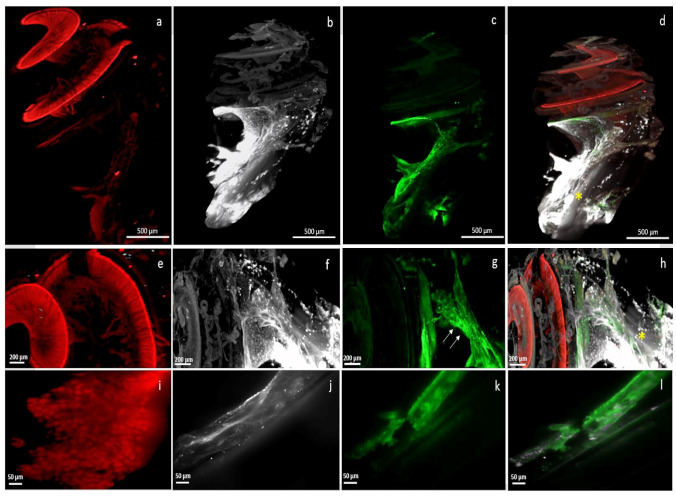
Right cochlea implanted with an electrode array and immunolabelled at low magnification, scale 500 µm (**a**–**d**) and at higher magnification, scale 200 µm (**e**–**h**) (4× objective); (**i**–**l**) are acquired with a 20× objective (scale 50 µm); (**a**,**e**,**i**) showed the labelling of type 1 afferent neurons of the spiral ganglion by NaKATPase alpha 3; (**b**,**f**,**j**) showed the proximal fibrotic reaction surrounding the alpha-SMA-labelled implant; (**c**,**g**,**k**) showed the macrophages present at the level of the implant, labelled with F4/80; (**d**,**h**,**l**) were merge images, highlighting the fluorescence gap (yellow asterisk).

**Figure 4:**
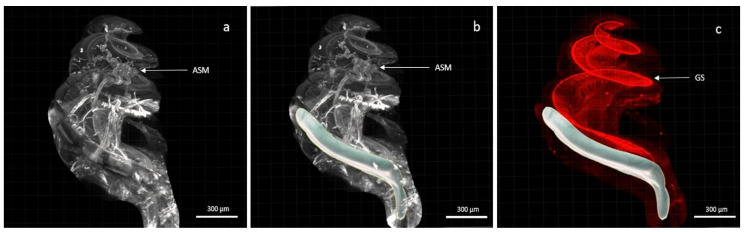
Cochlea with the electrode array in situ. (**a**,**b**) showed labelling of fibroblasts and arterioles by alpha SMA; (**c**) showed labelling of type 1 neurons in the spiral ganglion by NaKATPase alpha 3. In (**b**,**c**) the electrode array was modelled using Imaris software. (Scale 300 µm, 4× objective).

The electrode array was kept in situ throughout the clearing process (Figure 2) and imaging acquisition was performed with the electrode array in place, thus avoiding any loss of membrane and architectural integrity (Figure 3 and Figure 4). The electrode array was not labelled with antibodies, but the tissue response surrounding the implant was visible, labelled with alpha SMA (Figure 3). This fibrotic reaction was developed at the beginning of the basal turn of the cochlea and did not extend to the middle or apical turn and thus was related to the position of the electrode array (Figure 3). In addition, macrophages, labelled with F4/80, were present around the implant (Figure 3). Type 1 neurons in the spiral ganglion and their afferents to the hair cells were labelled with NaKATPase alpha 3, allowing the evaluation of neuronal changes (Figure 3). The resolution of the images acquired with the 20× objective allows a good identification of the cells (Figure 3).

**Figure 5:**
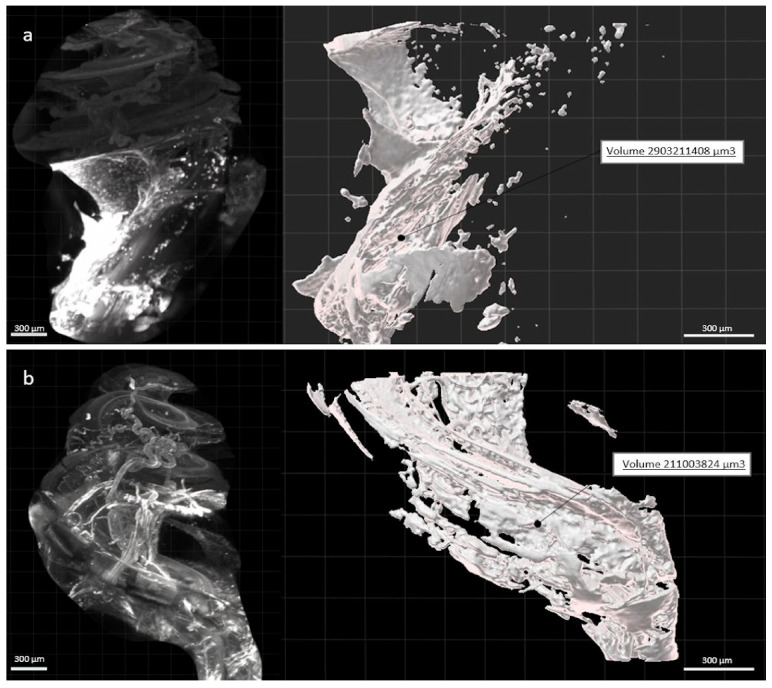
Image of a right cochlea implanted with a non-eluting (**a**) and dexamethasone-eluting (**b**) electrode array and their respective fibrosis volume created from the automatic surface creation wizard. Scale: 300 µm.

**Figure 6:**
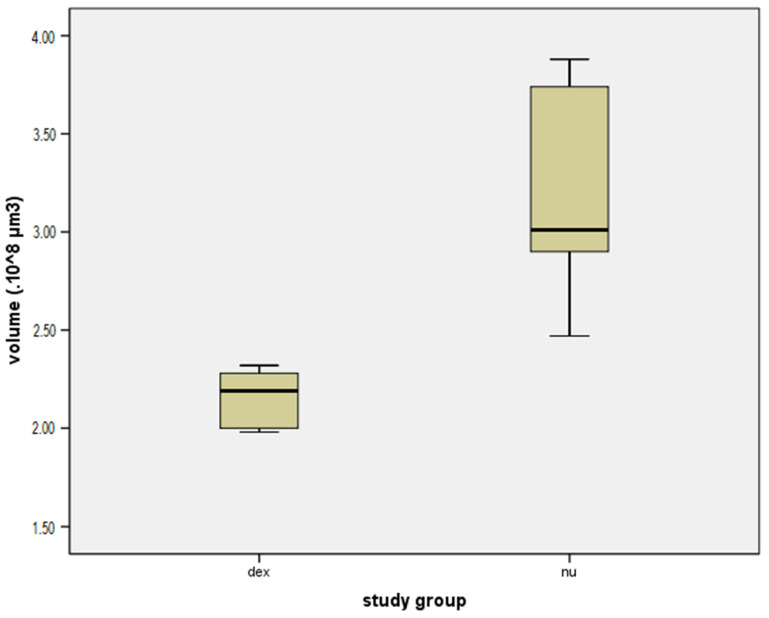
Box plot comparing the volume of local fibrosis (×$10^{8}{\;\mu m}^{3}$) in the dexamethasone group (dex) and the group without dexamethasone (nu).

Contouring of the fibrosis around the electrode array, using the creation wizard available in Imarisn (Figure 5) showed a mean volume of 2.16 × $10^{8}\;{\mu m}^{3}$ ± 0.15 in the dexamethasone group and 3.17 × $10^{8}\;{\mu m}^{3}$ ± 0.54 in the non-dexamethasone group. The difference between the groups was significant (*p* = 0.004) (Figure 6).

## 4. Discussion

Cochlear implantation can restore hearing in patients with significant hearing loss by electrically stimulating spiral ganglion neurons. However, the inflammatory response that occurs after cochlear implantation promotes the formation of fibrous tissue around the electrode array, which can negatively affect hearing outcomes after implantation [49,50]. Improvements in surgical techniques and cochlear implant insertion techniques have already made it possible to reduce postoperative trauma. Indeed, the benefit of round window implantation on the preservation of hearing thresholds and on structural damage is already well demonstrated [19,51]. Less invasive surgical approaches are being developed, such as the endomeatal approach or robot-based assistance, which ensure that the implants are inserted in an optimal axis, adapted to anatomical variations, while minimising trauma [20,52,53]. In addition, corticosteroid administration is considered an effective approach to reduce the inflammatory response and preserve residual hearing loss [35,54]. As systemic administration of corticosteroids has various side effects, local administration of dexamethasone has received considerable attention. Different delivery systems have already been proposed to determine the most effective and least invasive system, and further studies are still needed [35,43,55]. Light-sheet microscopy imaging is a suitable technique for the assessment of post-implantation damage and evaluation of drugs protecting the inner ear. Acquisition of the entire cleared cochlea by light-sheet microscopy allowed for accurate 3D reconstruction. The cochlear implant could be left in situ without interfering with the acquisitions. Immunostaining allows the study of specific components such as spiral ganglion neurons, macrophages, and fibrosis [46,47]. Alpha SMA labelling on arterioles and fibroblasts present during the healing process allowed us to observe the myofibrotic reaction around the electrode holder left in situ (Figure 3) [44,47]. The basal part of the cochlea is most affected by fibrosis and osteoneogenesis induced by cochlear implant surgery, and these results are consistent with analyses of other studies [7,44,56]. Our study shows that the post-implantation immune response can be reduced using a local dexamethasone-eluting implant, compared with a non-eluting implant. The volume of fibrosis surrounding the electrode array was significantly different in the two groups (*p* = 0.004). These results are consistent with other studies and support the efficacy of drugs administered locally to the cochlea [28,54]. Although the concentrations of dexamethasone in the perilymph were not measured here, it appears that the release of dexamethasone embedded in a silicone matrix would be continuous and prolonged [57,58,59]. A study evaluating the release kinetics of dexamethasone loaded in a silicone matrix showed continuous release for 90 days in the artificial perilymph, and this was confirmed in vivo over a 30-day follow-up period [57]. The electrode array as a vehicle for corticosteroid delivery to the inner ear thus appears to be an attractive option for inner ear protection after implant trauma and does not require an additional surgical procedure. Other teams have also tested the cochlear implant as an inner ear delivery system [14,36,60]. A dexamethasone-loaded hydrogel around the electrode was used and showed a decrease in fibrosis formation around the implant and an improvement in electrode impedances [14,61]. Comparison of implant impedance measurements between the two groups would be a clinically relevant endpoint to confirm our results.

Classical histological sectioning methods used to study the anatomy of the cochlea require sophisticated techniques and the implant must be removed [41,42]. Currently, confocal microscopy dominates studies of cochlear anatomy because of its high resolution and ability to visualise immunolabeled structures of interest, such as hair cells, spiral ganglion neurons [37,39,48,62]. Nevertheless, the acquisition of the whole cochlea in confocal microscopy is very difficult due to the long acquisition time that can lead to photobleaching of the samples and the difficulty of working with large samples [48,63]. To solve these problems, methods of dissection of the organ of Corti or removal of the bone capsule are used. Removal of the bone capsule allows preservation of the helical shape. Unfortunately, these sample preparation disrupted surrounding structures [40,48]. In addition, even implanted specimens can be imaged using confocal microscopy without big circumstances, but in some cases, the implant induces scattered radiation of the laser beam, and in addition, it is impossible to image through the CI which hinders the imaging of tissue that is behind the implant [12,48]. Although the resolution is lower than that of confocal laser scanning microscopy, light-sheet microscopy acquisitions are easy and allowed complete visualisation of the NaKATPase alpha 3 immunolabeled spiral ganglion (Figure 3). Acquisition with the 20× objective provides sufficient resolution to distinguish spiral ganglion neurons allowing cell counting analyses (although not per-formed here) and would thus allow the study of post-implantation neuronal cell loss without the disruption inherent in other sample analysis techniques. One study showed also that cochleae can still be sectioned after Spurr’s resin inclusion cleaning and imaging after light-sheet microscopy acquisition [47]. These results suggest that materials prepared for light-sheet microscopy can be examined by other microscopic techniques.

The presence of macrophages around the F4/80-labeled implant was also noted (Figure 3). A chronic inflammatory reaction characterised by long-term macrophage activity after the onset of cochlear injury has already been demonstrated [44]. Volumetric analyses can be used to quantify macrophages in regions of interest in the cochlea [47]. Immunostaining of hair cells was not performed but would similarly allow the analysis of the entire organ of Corti without loss of surrounding structures [46]. Some teams have already developed cell counting methods from images acquired by light-sheet microscopy [46]. It will be interesting to be able to apply these methods to our samples in order to obtain information on cell survival because inflammation related to cochlear implant surgery promotes the loss of remaining spiral ganglion neurons and auditory hair cells that are crucial for auditory perception [1,17,18,64]. In addition, preservation of residual hearing is important for enabling the use of cochlear implants devices with combined electrical and acoustic stimulation [17,18].

Among the limitations of this study, it is important to note that it was performed on a small number of animals and that it will be necessary to verify the reproducibility of the results on a larger sample. Moreover, objective quantification of cells damages would be important to complete the evaluation of this dexamethasone delivery system. Finally, this study does not account for clinically relevant criteria such as preservation of auditory thresholds or measurement of impedances that are directly correlated to the degree of post-implantation trauma. 

## 5. Conclusions

The use of the implant itself as a galenic for corticosteroid administration appears to be an attractive option for inner ear protection after implant trauma. Our results on the reduction of fibrosis reaction around the electrode array are encouraging. The three-dimensional immunofluorescence analysis technique allows for the study of post-implantation damage in both tissues and cells in the same sample.
